# Editorial: CardioNeurology: Basic, translational and clinical research

**DOI:** 10.3389/fcvm.2022.1114419

**Published:** 2023-01-04

**Authors:** Leonardo Roever, André Rodrigues Durães, Octávio M. Pontes-Neto

**Affiliations:** ^1^Department of Clinical Research, Federal University of Uberlândia, Uberlândia, Brazil; ^2^Programa de Pós-Graduação em Medicina e Saúde-Universidade Federal Da Bahia, Salvador, Brazil; ^3^Stroke Service, Neurology Division, Ribeirão Preto Medical School, University of São Paulo, Ribeirão Preto, Brazil

**Keywords:** CardioNeurology, stroke, heart disease, risk factors, mortality

Recent advances are allowing a better understanding of the interaction between the heart and the brain. Many acute cardiovascular events require a detailed neurological evaluation, and vice versa. With the advancement of knowledge, studies have allowed us to verify more points of connection between stroke, dementia, cerebral small vessel disease and its relationship with congestive heart failure, myocardial infarction, atrial fibrillation (AF), and other cardiovascular conditions (1).

In this Frontiers Research Topic, an international selection of high-quality studies has contributed to advancing our understanding of CardioNeurology, both in the field of diagnosis, treatment and imaging technologies. We believe that this edition about such a hot topic will become a valuable source of information for clinicians and researcher in the field.

To illustrate this view, Ernst et al. presented a study on whether low heart rate variability could predict stroke and other complications after hip surgery. Follow-up was 6 months and findings were related to mortality, pneumonia, stroke, and myocardial infarction (Ernst et al.). Zhou et al. studied about gender and age-specific associations and visit-to-visit blood pressure variability with anxiety. In their studies that included 48,023 patients, women were more likely to develop anxiety and among the predictors advanced age, cardiovascular, metabolic, and gastrointestinal diseases stood out (Zhou et al.). Naranjo et al. in a cohort of 245 ischemic strokes evaluated the presence of 28% of antiphospholipid antibodies (aPL) with an OR 2.40. Other risk factors such as arterial hypertension, atrial fibrillation, and active smoking have also been identified (Naranjo et al.).

In addition, Risseeuw et al. presented a case of a 69-year-old woman with a history of cigarette smoking, arterial hypertension, type 2 diabetes mellitus, hypercholesterolemia, right subcapital femur fracture, with repetitive episodes of Takotsubo syndrome (TTS). In this case the authors observed the link between neurological disease and TTS, especially with involvement of the medulla oblongata (Risseeuw et al.). Teodoro et al. evaluated whether the percentage of patients with undetermined etiology according to the TOAST classification decreased after transthoracic echocardiography. In a total of 1,100 patients, echocardiography reduced the odds of an indeterminate TOAST score and the risk of in-hospital mortality (Teodoro et al.). In the retrospective study by Long et al. with 7,528 septic patients. The presence of a coagulation disorder in the first 24 h after admission to the ICU was found to be an independent risk factor for AF and its 90-day mortality varies with the severity of the clotting disorder (Long et al.).

Another study by Lu et al. with 296 patients the incidence of bleeding events in AF patients treated with ticagrelor was comparable to that in patients treated with clopidogrel plus aspirin at 6 month. In another study in Hong Kong, Mui et al. demonstrated that the use of sodium-glucose cotransporter 2 inhibitors is associated with lower risks of dementia, Parkinson's disease, and cerebrovascular mortality compared with dipeptidyl peptidase-4 inhibitors in patients with type 2 diabetes mellitus.

In a Bayesian Network Meta-Analysis, Duan et al. analyzed adequate time from collapse to return of spontaneous circulation (ROSC), which is an ideal indication for targeted temperature management (TTM) to improve survival and neurological outcome. The authors found that survival and good neurological outcome are closely associated with the time to collapse for ROSC (Duan et al.). In another study Zhang et al. investigated the acute hemodynamics of the lower extremities during enhanced external counterpulsation. Counterpulsation acutely improved blood flow, blood flow velocity, and maximum systolic accelerations of the anterior tibial artery. As well as significantly increased blood flow velocity and peripheral resistance of the inferior knee artery, while markedly reducing blood flow in the posterior tibial artery (Zhang et al.).

In an experimental model Park et al. found that reducing expression of the huntingtin gene mutation in the heart benefits cardiovascular function in the BACHD Mouse Model of Huntington's Disease. In the study by Primessnig et al. found that B-type natriuretic peptide (BNP) improves diastolic tension during adrenergic stress in human atrial myocardium and may have long-term positive effects on inotropic reserve. And that BNP and Sacubitril + valsartan can reduce atrial arrhythmogeneity during adrenergic stress *in vitro* (Primessnig et al.).

In the case study Moreno et al. demonstrated that non-compaction cardiomyopathy (NCCM) is associated with neuromuscular disorders. Patients with NCCM with a phenotype of dilated left ventricle with reduced left ventricular ejection fraction must be treated promptly to avoid the progression of heart failure (Moreno et al.). Another study by Zhu et al. demonstrated an expert consensus on the use of coated stents to treat complex cerebrovascular diseases. Other authors have shown that covered stents are an effective treatment option for complex cerebrovascular diseases, such as complex aneurysms, direct carotid-cavernous fistulas and internal carotid artery injuries (Zhu et al.).

In conclusion, the innovations presented in this valuable topic were fundamental to further strengthen and clarify the interrelationship between the cardiovascular and cerebral segments. Undoubtedly, they are extremely complex and intertwined systems. A holistic and complementary view of cardiology and neurology is essential for a more complete management of patients affected by such pathologies.

## Author contributions

LR, AD, and OP-N contributed to the conception, design, and drafting of the work. All authors contributed to the article and approved the submitted version.
